# High-throughput screening to identify inhibitors of the MTB CARD•RNA polymerase protein-protein interaction

**DOI:** 10.3389/ftubr.2026.1937953

**Published:** 2026-10-02

**Authors:** Glory M. Velazquez, Jonathan Z. Sexton, Scott G. Franzblau, Gauri S. Shetye, Akil Hossain, George A. Garcia

**Affiliations:** 1 Department of Medicinal Chemistry, University of Michigan, Ann Arbor, MI, United States; 2 Institute for Tuberculosis Research, University of Illinois, Chicago, IL, United States

**Keywords:** antibiotic discovery, CarD, fluorescence polarisation, high through put screening, Mycobacterium tuberculosis, protein protein interaction (PPI), RNA polymerase (RNAP)

## Abstract

In 2023, tuberculosis (TB) once again became the world's deadliest infectious disease, overtaking COVID-19 as the leading cause of death from a single infectious agent, Mycobacterium tuberculosis (MTB). The World Health Organization (WHO) reports that the COVID-19 pandemic has reversed years of progress in providing essential TB services and reducing TB disease burden. In 2025, TB was responsible for an estimated 1.23 million deaths globally (95% uncertainty interval: 1.13–1.33 million), and there were 400 K cases of rifamycin resistant/multidrug resistant TB (RR, RIFR, MDR). While treatment success for drug susceptible TB remains high (88%), outcomes for MDR/RR-TB are lower but improving, reaching 71% globally. Clearly, there is a critical unmet need for novel antibiotics against MTB, exacerbated now due to COVID-19. The limited success in pursuing novel targets underscores the urgent need to revisit and reevaluate the mechanisms of action behind existing and once effective drugs, a strategy that may hold untapped potential in the fight against growing antibiotic-resistant TB. The MTB RNA polymerase (RNAP) is a proven target for TB treatment by rifampin (RMP), which kills both active and latent MTB. However, RMP suffers from several drawbacks including: long treatment times, activation of hPXR (inducing Cyp3A4 which is problematic for HIV-TB coinfection), and resistant mutations within RNAP. Two newer treatments with novel mechanisms of action include bedaquiline and delamanid, the former has been approved by the FDA for the treatment of multiple-drug resistant tuberculosis (MDR-MTB); however, even these new drugs already have incidences of acquired resistance. Our *goal* is to develop novel drug candidates for TB treatment that: target RNAP, have the potential to shorten treatment times, and have activity against RIF^R^ mutants. Our approach, orthogonal to the rifamycins, is to target the interaction of MTB RNAP with the required transcription factor CarD. We have previously labeled CarD with the BODIPY-FL fluorophore via engineered cysteine mutations and developed a fluorescence polarization assay to monitor its interaction with RNAP and promoter DNA. Leveraging this target, we screened a 24 K small molecule library against CarD binding to MTB RNAP. We have now extended this screening to∼88,000 additional molecules.

## Introduction

The World Health Organization (WHO) estimates that a total of 10.7 million people developed TB in 2025 globally and 1.23 million people died from tuberculosis disease (TB) in 2025 (1). Rifampin (RMP) revolutionized the treatment of tuberculosis and has been the cornerstone of regimens since the 1980's (1). Including RMP in therapeutic regimens reduced treatment time from 18 months to 6–9 months. These treatment plans usually include isoniazid (INH), which inhibits cell wall biosynthesis (2) and a combination of pyrazinamide and ethambutol (2, 3). Nevertheless, resistance to current frontline therapeutics for the treatment of tuberculosis represents a critical threat to human health. In 2021, 410,000 people were diagnosed with MDR/RR-TB, a disease state which is characterized by resistance to RMP and INH, and 27,075 were reported to have extensively drug resistant TB (XDR-TB) (4). XDR-TB maintains the same resistance characterization as MDR-TB with the addition of resistance to any fluoroquinolone and at least one aminoglycoside (4, 5). Additionally, RMP suffers from several drawbacks including: long treatment times, activation of hPXR (inducing Cyp3A4 which is problematic for HIV-TB coinfection), and resistant mutations within RNAP (6, 7). Two newer drugs, bedaquiline and delamanid, are novel therapeutics being used for the treatment of MDR-TB (8, 9). However, acquired resistance, from mutations in the HTS transcriptional regulator *mmpR*, to bedaquiline has already been observed (10, 11). Clinical data also indicate that bedaquiline-resistant tuberculosis is associated with delayed microbiological responses and poor treatment outcomes, underscoring the continued need for therapeutics directed toward mechanistically distinct targets (12, 13). Delamanid has been approved for use by the European Medicines Agency (EMA), but has yet to receive authorization from the FDA. Since 2000, only 5 first-in-class antibiotics have been launched, none of which target MTB (12). The lack of new antibiotics and novel targets poses a serious unmet gap which needs to be addressed.

The MTB RNA polymerase (RNAP), potently inhibited by rifampin, is a proven target due to its essentiality for active and persistor infections and the low similarity between bacterial and eukaryotic RNAPs (15). After RNAP binds to a promoter, forming the closed complex (RPc), the transcription bubble/open promoter complex (RPo) is formed by unwinding the DNA allowing RNA to be transcribed (Figure 1A) (16). This step is thought to be the rate limiting step for gene expression (17). CarD is a global transcriptional regulator found in certain groups of both gram positive and gram negative bacteria (18). CarD binds to the RPo and stabilizes the open promoter complex, resulting in increases in transcription from promoters with short-lived RPos and decreases in transcription from promoters with long-lived RPos (Figure 1B) (16, 19, 20). Stabilization of this complex is essential for MTB viability; attempts to remove CarD from the MTB genome were lethal (21). However, conditional depletion of CarD using a tetracycline-controlled gene expression led to increased sensitivity to reactive oxygen species and RMP, as well as the inability of MTB to replicate and persist in mice (21, 22). Many of the mutations in CarD which were attempted to be introduced through homologous recombination were not tolerated which suggests that small changes in protein structure have a great impact on CarD function and MTB viability (21, 23). Therefore, therapeutics which prevent CarD association with RNAP could show efficacy against MTB. Recent structural studies have provided additional insight into the dynamic nature of MTB transcription initiation. Cryo-electron microscopy studies have captured multiple transcription-initiation and promoter-escape intermediates and have revealed conformational rearrangements of RNAP associated with promoter escape and early RNA synthesis. Importantly, recent structures of MTB transcription complexes include CarD and provide additional structural context for its role within the transcription-initiation machinery (24, 25). CarD homologues are present in many bacterial phyla (disease states) including: *Chlamydiae* (chlamydia), *Firmicutes* (*C. diff.* colitis), and *Spirochaetes* (Lyme disease) (18, 21). With this in mind, it is our belief that CarD represents an appealing target for therapeutic discovery for the treatment of tuberculosis as well as other clinically relevant pathogens.

**Figure 1:**
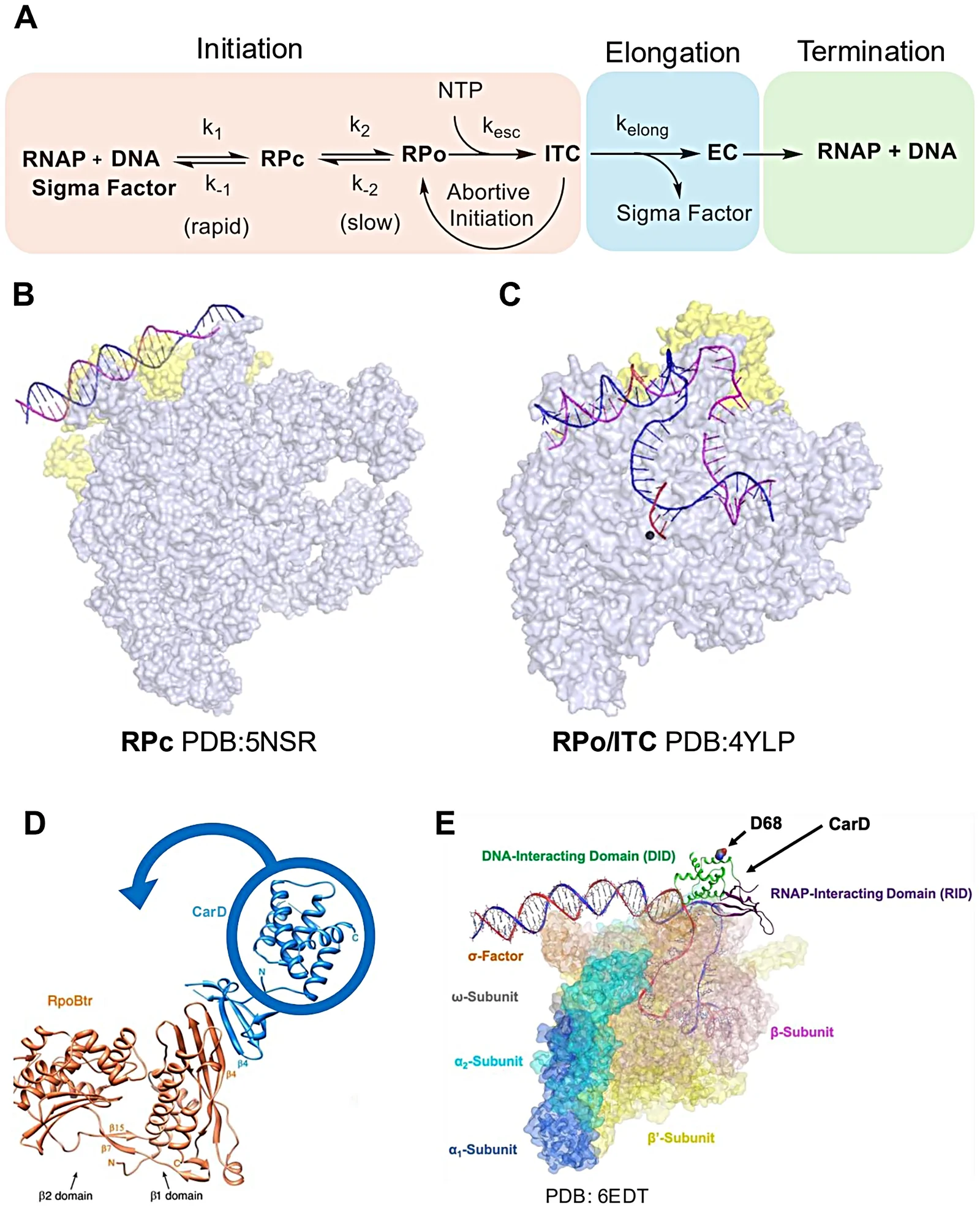
RNAP kinetics and structures of MTB RNAP, CarD and complexes. **A**) RNAP. DNA closed complex (Rpc) (14), **B**) RNAP•DNA open complex (Rpo) (14), **C**) CarD bound to RNAP β subunit, **D**) CarD bound to Rpo, **E**) CarD (pink) with potential small molecule binding sites determined by MOE site finder (grey and red surfaces). Figure 1E taken from Stefan et al. (25).

The repurposing of drugs, which involves applying existing therapeutics to new diseases, holds the potential for a rapid clinical impact at a reduced cost compared to *de novo* drug development. Development timelines for repurposed drugs average 3–12 years, compared to 10–17 years for novel compounds, and costs are dramatically reduced—about $300 million on average vs. $2–3 billion for new drugs. The probability of regulatory approval for repurposed drugs is approximately 30%, which is three times higher than the typical 10% success rate for new molecules, due largely to existing safety data and established clinical experience. Drug repurposing, therefore, represents an attractive and cost effective option for accelerating drug discovery and reducing attrition rates (26). However, limitations such as the absence of a definitive physical drug collection and insufficient readouts of drug activity for predicting new indications have limited the systematic repurposing efforts (26). Still, efforts have been made to perform drug repurposing screenings for life threatening diseases such as COVID-19 and Colorectal cancer (CRC) (27, 28). These efforts highlight both the promise and the ongoing need for more systematic, target driven approaches to fully realize the potential of drug repurposing.

We have developed, validated and utilized a fluorescence polarization assay, where CarD binding to the RNAP•DNA complex can be monitored using BODIPY-FL labeled CarD (CarD-BODIPY-FL, green fluorescence) (29). Since both the RNAP and DNA interactions are required for highest affinity binding to CarD (Figure 1), both the C-terminal and N-terminal domains of CarD and the linking interface are potential targets. We used this assay to screen the Maybridge 24 K compound library for small molecule inhibitors of CarD•RNAP association (29). From this screen, we identified one hit compound with relatively weak activity (IC_50_ ∼150 μM). Examination of the CarD structure has revealed several potentially druggable pockets in the C-terminal domain of CarD, several of which are adjacent to residues shown to be critical for interaction with promoter DNA as well as in the linking region between the CarD N- and C-terminal domains. Small molecule binding in the interdomain pockets may prevent conformational movements required for CarD function.

Overall, CarD represents a critical target for treatment against tuberculosis, and efforts have been made to identify potential inhibitors of the interactions between CarD and the RNAP·DNA complex. The long-term goal of this project is to identify molecules capable of dysregulating RNA transcription in mycobacteria. The overall objective is to identify and optimize therapeutic molecules with potent, selective, inhibitory effects against the CarD•RNAP interaction to eliminate MTB cells during infection, whether active or latent.

Here, a 4719-compound drug repurposing library was screened using our CarD FP assay. Molecules with varying activity were identified and analyzed. In this study, we included a repurposing collection as a comparative screening set, while focusing primarily on a large, diverse small-molecule library to identify novel inhibitors of the CarD•RNAP interaction. This screen was performed with a library of 82,516 small molecules, cherry-picked for drug-like properties from the Dart Neurosciences collection. Several potential hits were re-confirmed following the primary screen, and their potential inhibitory activities were further characterized.

## Results

### Drug repurposing library screen

A collection of 4,719 compounds from a drug repurposing library was screened at 20 μM to provide a benchmark for assay performance and to identify potential reference inhibitors (Figure 2) (30). While several compounds initially exhibited apparent inhibition, a substantial fraction showed fluorescence interference, complicating interpretation (Supplementary Figure 1). After filtering for interference free signals, only a small number of compounds met the hit criteria. One of the hit compounds was fidaxomicin (not shown), a known inhibitor of DNA binding to RNAP that we previously characterized as indirectly inhibiting CarD binding to RNAP (29). It is worth noting that our screen did not identify rifampin, a potent inhibitor of RNAP that does not affect CarD binding to RNAP. These results help to validate our screen.

**Figure 2:**
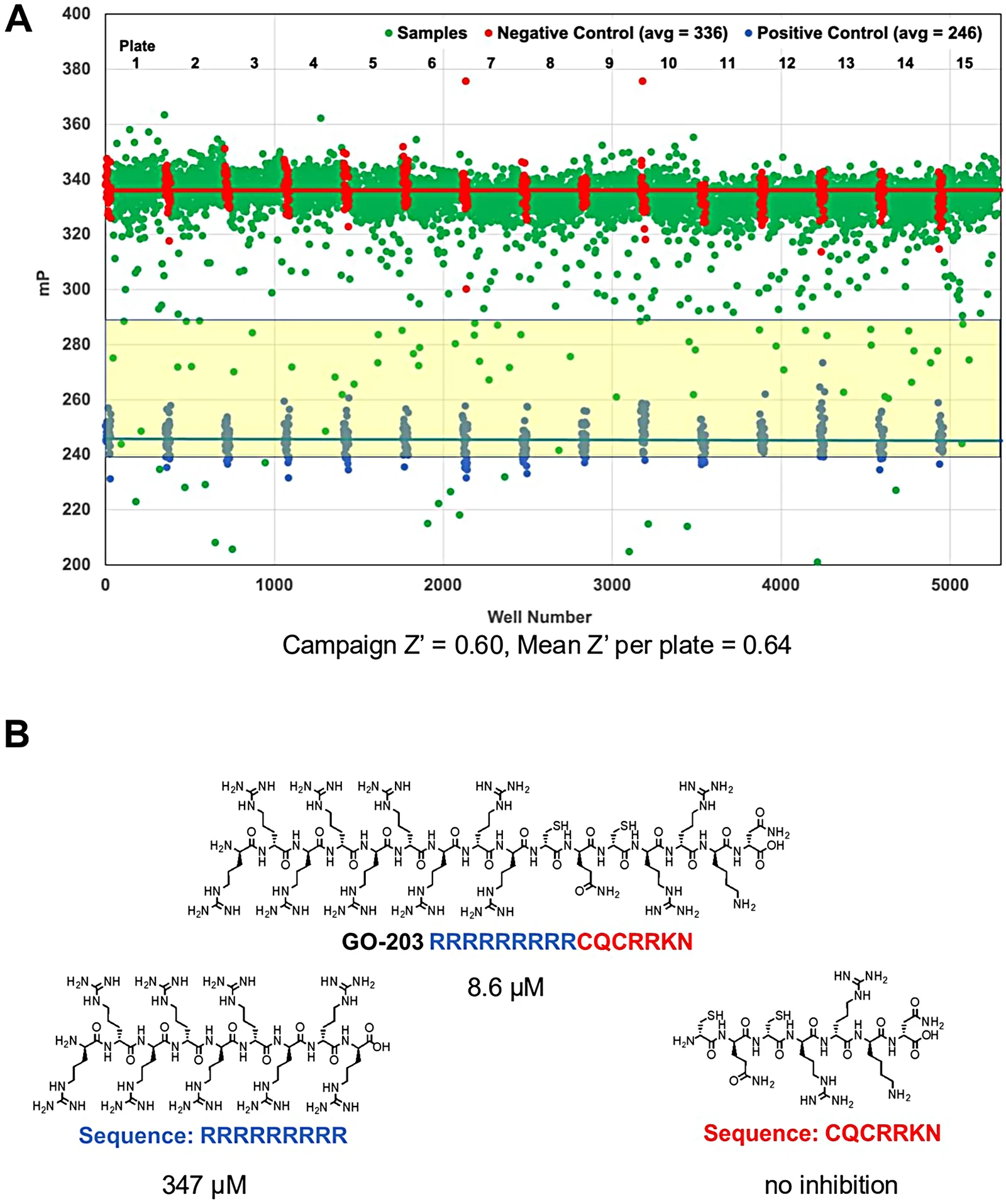
Drug repurposing library screen results. **A**) HTS results (the average Z-factor per plate=0.64), **B**) GO-203 hit and component peptides.

### Reconfirmation

Fresh powder samples of each hit molecule were purchased to determine IC_50_ curves within the concentration range of 20–100 µM. Among the compounds initially identified as active, GO-203 was selected for follow up analysis due to its apparent potency in the primary assay. However, further characterization revealed atypical dose–response behavior and significant fluorescence related artifacts. To clarify its mechanism, we evaluated peptide fragments corresponding to its functional domains and performed fluorescence intercalator displacement (FID) assays (Figure 2 and Supplementary Figure 2). FID is a convenient and practical tool for identifying nucleic acid binding ligands. This assay is based on the loss of fluorescence of a DNA bound intercalator, such as ethidium bromide (EtBr). To perform the FID, we used a duplex DNA oligonucleotide called LacUV5, a form of the lac promoter found in *E. coli.* These studies demonstrated that GO-203 and its R9 containing fragment interact with DNA, indicating that the observed inhibition arises from DNA binding rather than direct disruption of the CarD•NAP interaction. This finding highlights a key limitation of the assay format, in which compounds that perturb DNA can produce apparent inhibitory signals. Taken together, results from the repurposing screen indicate that compounds identified in this format can be associated with indirect or assay interfering mechanisms, underscoring the need for larger and more diverse libraries coupled with rigorous follow-up assays. Therefore, we next focused on screening the DART Neurosciences collection to identify novel small molecules with improved specificity.

### DART neurosciences library screening

A screen of 82,516 compounds, each tested at a concentration of 20 µM, from the *DART Neurosciences* collection was conducted (Figures 3, 4). The entire screen Z-factor was 0.62, and the average Z-factor per plate was 0.78. As part of the criteria to filter hit compounds, hits were considered as those compounds which had mP values 3 standard deviations lower than the negative control and 3 standard deviations higher than or equal to the positive control (1,242 hits). Compounds exhibiting activity greater than 110% relative to the positive control, on a plate-by-plate basis, were excluded from the hit pool (1,189 hits). Additionally, compounds with a parallel intensity exceeding 100,000 were also eliminated (1,183 hits). Any compounds that were flagged (e.g., toxicity, chemically reactive, promiscuous/PAINS) were taken out as well (1,054 hits). Promiscuity was assessed by the CCG using internal screening data, where compounds with a high frequency of activity across diverse assays were deprioritized. PAINS flags were assigned computationally through MScreen based on substructure filters defined in the literature, with compounds categorized as yellow, red, or black based on the predicted severity of their interference potential.

**Figure 3:**
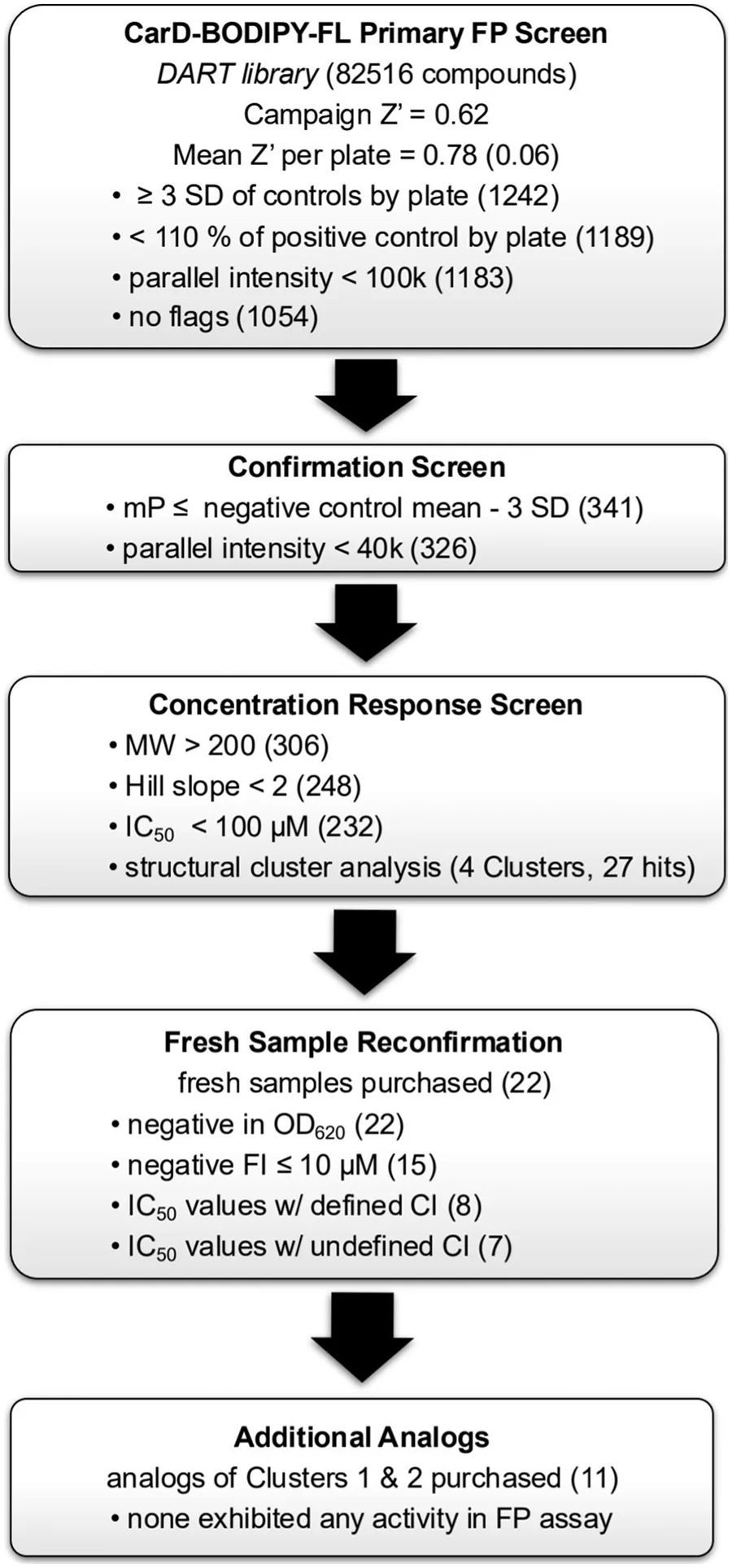
DART library screen flowchart.

**Figure 4:**
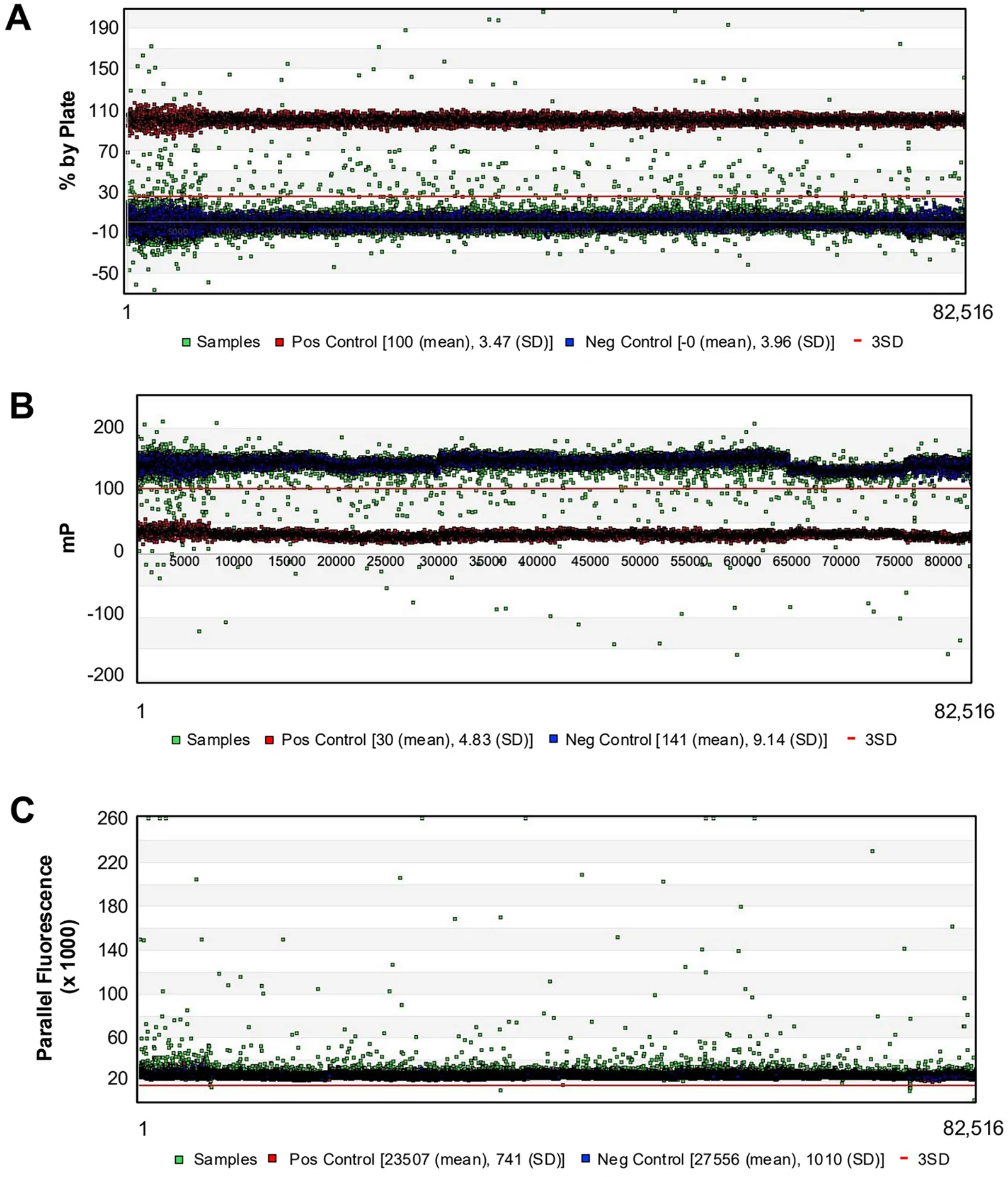
DART library screen results. **A**) HTS results (the average Z-factor per plate=0.78), **B**) Initial hit compounds with their percent inhibition at 20 µM, **C**) Parallel fluorescence intensity.

Hits were reconfirmed, and a counter screen was performed to detect potential false positives due to fluorescence interference. This involved incubating the compounds with CarD/RNAP and reading the plate prior to adding DNA. Compounds that had an mP value of less than three standard deviations of the negative control in the reconfirmation screen progressed (341 hits). Next, compounds were filtered based on their fluorescence interference. Any compounds that exhibited parallel intensity larger than 40,000 (upper limit for acceptable background fluorescence) in the counter screen were removed, leaving 326 hits to progress to concentration response evaluation. The threshold was established empirically by visual inspection of fluorescence-intensity distributions across reconfirmed hits and comparison of intensity profiles with the corresponding FP concentration-response curves.

Concentration response curves (CRC) were determined for the hits. Compounds with molecular weight > 200 (306 hits), Hill slopes < 2 (248 hits), and IC_50_ < 100 µM (232 hits) progressed to structural Cluster analysis. A Cluster analysis was performed, and seven clusters were highlighted with average pIC_50_ values > 4.5 (IC_50_ ∼30 µM) and 204 compounds were singletons. Fresh samples for 22 compounds from four clusters were ordered for reconfirmation based on their IC_50_ (<60 µM).

### Reconfirmation

Concentration response curves were determined for the 22 fresh samples to confirm the results obtained using the HTS stocks (Supplementary Figure 3). A notable challenge was the significant fluorescence interference (FI) observed for many compounds [Table 1, FI @ (µM)]. To mitigate this issue, IC_50_ values were calculated using data from concentrations where no FI was detected (Supplementary Figure 3). Eight compounds yielded IC_50_ values with determined confidence intervals (CI). Seven compounds yielded IC_50_ values, however, their confidence limits were undefined. Six compounds with excessive FI were excluded from further analysis. One compound had no activity at concentrations below its FI threshold. All compounds also exhibited Hill slopes between -1and −2, with three exceptions (−0.2, −2.2, and −2.4). Subsequently, we procured 11 analogs of compounds from Clusters 1 and 2. However, none of these analogs exhibited significant activity and were, therefore, not assessed in secondary assays. Both the initial hits and analogs showed fluorescence interference above certain concentrations, which, while not necessarily indicative of a lack of activity, complicated the accurate assessment of their activity. While fluorescence interference was observed during reconfirmation of DART hits, IC₅₀ values were calculated using concentration ranges where interference was minimal. Importantly, orthogonal assays, including FID and *in vitro* transcription, did not support DNA binding or RNAP inhibition for these compounds, providing supporting evidence for a mechanism distinct from DNA- or RNAP-mediated effects.

**Table 1:** DART screen Hit molecules activity data.

Compound ID	HTS Stock CRC (CarD FP Assay)		Reconfirmation CRC (CarD FP Assay)			Cytotoxicity	MIC_90_ (µg/mL)			MBC_99_ (µg/mL)
	IC_50_ (µM)	Hill Slope	IC_50_ (µM)	Hill Slope	FI @ (µM)	Vero Cell TC_50_ (µM)	MABA	LORA	LORA/MABA	
1-a	29	−1.6	25 (^a^)	−0.9	10	ND	65	ND	ND	>100
1-b	21	−1.5	7 (± 2)	−1.7	5.6	ND	>100 (38%)	ND	ND	>100
1-c	26	−1.0	36 (± 13)	−1.1	30	ND	>100 (37%)	ND	ND	>100
1-d	15	−1.7	117 (^a^)	−1.0	59	ND	>100 (26%)	ND	ND	>100
1-e	17	−1.9	25 (^a^)	−1.1	10	ND	69	ND	ND	>100
1-f	39	−1.2	30 (± 25)	−0.9	18	ND	60	ND	ND	>100
1-g	31	−1.1	NA		33	ND	>100 (17%)	ND	ND	>100
1-h	33	−1.5	Fluorescence Interference			ND	90	ND	ND	>100
2A-a	52	−1.0	23 (^a^)	−1.5	32	ND	97	ND	ND	>100
2A-b	35	−1.2	Fluorescence Interference			ND	56	ND	ND	>100
2A-c	48	−1.0	38 (^a^)	−1.1	18	ND	>100 (20%)	ND	ND	>100
2A-d	51	−1.2	33 (± 9)	−1.4	59	ND	>100 (20%)	ND	ND	>100
2B-a	17	−2.0	1.6 (± 1.4) mM	−1.1	2 mM	>100	43	>100 (38%)	NA	> 100
2B-b	44	−1.9	320 (^a^)	−0.2	33	14	57	52	0.93	> 100
2B-c	40	−1.2	17 (± 3)	−1.1	18	>100	67	>100 (0%)	NA	> 100
2B-d	21	−2.4	65 (^a^)	−2.4	33	24	1.4	79	55	47
3-a	7.2	−1.7	Fluorescence Interference			ND	64	ND	ND	>100
3-b	56	−1.0	Fluorescence Interference			ND	>100 (59%)	ND	ND	>100
3-c	37	−1.4	Fluorescence Interference			ND	>100 (20%)	ND	ND	>100
4-a	9.8	−1.7	38 (±17)	−2.2	30	ND	52	ND	ND	>100
4-b	7.9	−1.4	412 (±125)	−1.0	35	ND	>100 (10%)	ND	ND	>100
4-c	8.3	−1.7	Fluorescence Interference			ND	>100 (37%)	ND	ND	>100

ND, not determined, NA, no activity < FI Threshold.

a Confidence limit undefined.

> 100 (% inhibition @ 100 µg/mL).

### Fluorescent intercalator displacement (FID) analysis

The compounds were tested across a range of concentrations, beginning at 200 µM with subsequent serial two-fold dilutions (Supplementary Figure 4 and Table 1). Notably, none of the compounds caused a reduction in ethidium bromide fluorescence, indicating that none were able to displace the DNA. Some compounds exhibited minor interference with the ethidium bromide fluorescence signal, a challenge we had previously encountered with the fluorescence polarization (FP) assay.

### RNAP *in vitro* transcription inhibition counter screen

It is possible that compounds could bind to RNAP in such a manner to block DNA binding and consequently inhibit CarD binding. If this were the case, the compounds would certainly inhibit *in vitro* transcription by RNAP. Therefore, to identify inhibitors of MTB RNAP, an *in vitro* plasmid-based transcription assay that utilizes malachite green (MG) to detect transcribed RNA containing MG aptamers was performed with the compounds (Supplementary Figure 4 and Table 1). In this assay, RNAP transcribes pMGA12 (plasmid), resulting in RNA transcript containing 12 repeats of the MGA sequence. These aptamers subsequently fold into their secondary structures, enabling binding to MG yielding a large increase in MG fluorescence. Compounds were tested starting at 200 µM with subsequent serial two-fold dilutions. None of the compounds inhibited RNAP activity.

### Minimum inhibitory concentration (MIC_90_) and minimum bactericidal concentration (MBC_99_)

The antibacterial activity of the 22 tested compounds was assessed using MIC₉₀ and MBC₉₉ determinations (Table 1). MIC₉₀ values ranged from approximately 56 µg/mL to >100 µg/mL, with compounds such as 2A-b (MIC₉₀ = 56.04 µg/mL) and 1-f (MIC₉₀ = 60.04 µg/mL) showing relatively higher potency compared to others. The MIC₉₀ values of several compounds, including 1-b, 1-c, 1-d, and 1-g, were reported as > 100 µg/mL, often accompanied by the percentage inhibition observed at the highest concentration tested, 100 µg/mL (e.g., 1-b: >100 µg/mL with 62% inhibition). Despite variations in MIC₉₀, the MBC₉₉ values for nearly all compounds were consistently >100 µg/mL, indicating limited bactericidal effects at the concentrations tested.

### Vero cell toxicity

Vero cell toxicity determinations were only made for those compounds with significant inhibitory activity (e.g., compounds 2B-a through d, Table 1). The Vero cell TC_50_ values for 2B-a, -b and -d are lower than their CarD•RNAP inhibitory activities, indicating a significant toxicity that will need to be addressed. However, it is encouraging that the MABA MIC_90_ for 2B-d is well below (5.6-fold) its Vero cell TC_50_.

### Structure activity analyses

Cluster 1 has a 5,6,7,8-tetrahydropyrido[3,4-*d*] pyrimidine connected through a methylene group to a 4*H*-pyrido[1,2-*a*] pyrimidin-4-one core structure. Modifications of the core structure primarily focus on altering the size and shape of the indicated substituents (R_n_) attached to the aromatic rings. This will impact the steric and electronic properties, and ultimately their efficacy as reflected in the IC_50_ values (Table 1). When the core molecule, 1-a (center), is substituted with methyl groups at the R_1_ and R_2_ positions (1-e), or only at the R_3_ position (1-c), the IC_50_ value is the same or close to 25 µM (Figure 5). A chlorine (Cl) at the R_2_ position with methyl at the R_1_ position (1-f) similarly does not change the IC_50_. If the size of the R_1_ substituent is increased, as seen in 1-d, this increases the IC_50_–117 µM. Modifying the pyrimidine ring to a carbocyclic ring, seems to also be detrimental to the activity, as observed with 1-j. However, a substantial improvement in potency is observed when only a methyl group is located at R_2_ position as shown in 1-b. This substitution lowers the IC_50_ approximately three-fold to 7 µM.

**Figure 5:**
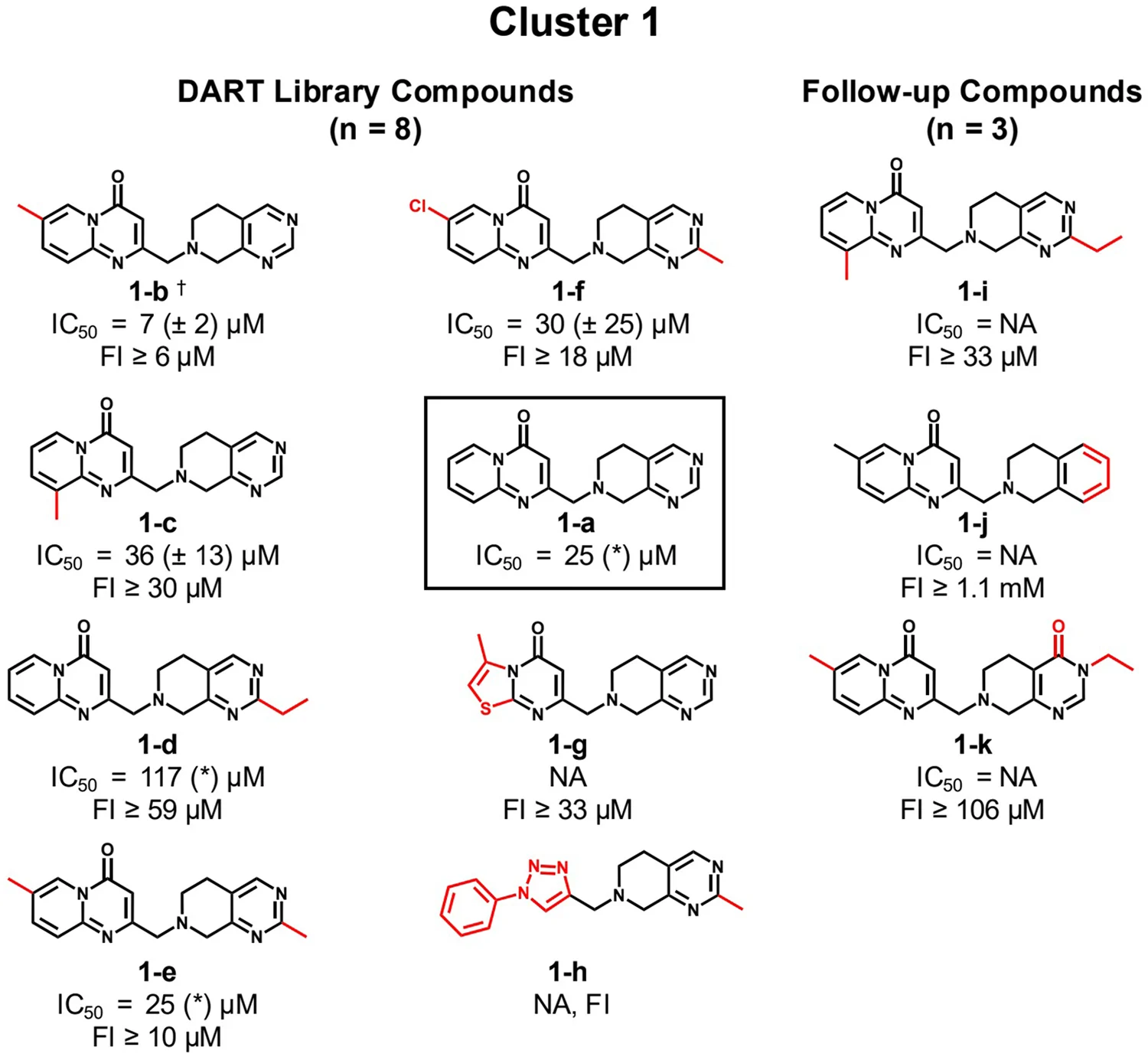
Structures of compounds from cluster 1. The core scaffold is in black, the varied substituents are in red. The * symbol represents an undefined confidence interval (CI).

Cluster 2 core structure contains a bicyclic 5,6,7,8-tetrahydropyrido[4,3-c] ring and pyridazin-3(2H)-one ring with a second bicyclic ring system attached (Figures 6, 7). The difference between Cluster 2A and 2B is the presence of a methylene group between the two rings in Cluster 2A, with consequent increases conformational flexibility over Cluster 2B (Figures 8, 9). Based on the IC_50_ values of most molecules from Cluster 2B, there appears to be a lack of tolerance to the more conformationally restricted heterocyclic rings. Compound 2B-c shows the highest potency with an IC₅₀ of 17 μM (Table 1), and this could be due a reduction in the size of the heterocycle. In Cluster 2A, 2A-a and 2A-c also demonstrated relatively strong inhibition with IC₅₀ values of 23 μM and 38 μM, respectively. Compound 2A-b, which has a similar structure to that of 2A-c, displayed excessive fluorescence interference. Considering that the only change that could be attributed to this interference is the replacement of ethyl to a methyl in position R_1_, it makes it hard to establish a consistent trend between the structure and activity. Overall, more information is needed to properly predict what structural changes can improve the affinity of these compounds. Unfortunately, the analogs from this Cluster also showed too much fluorescence interference to be properly analyzed. Some potential reasons for this case would be the presence of extended conjugated aromatic and heteroaromatic systems.

**Figure 6:**
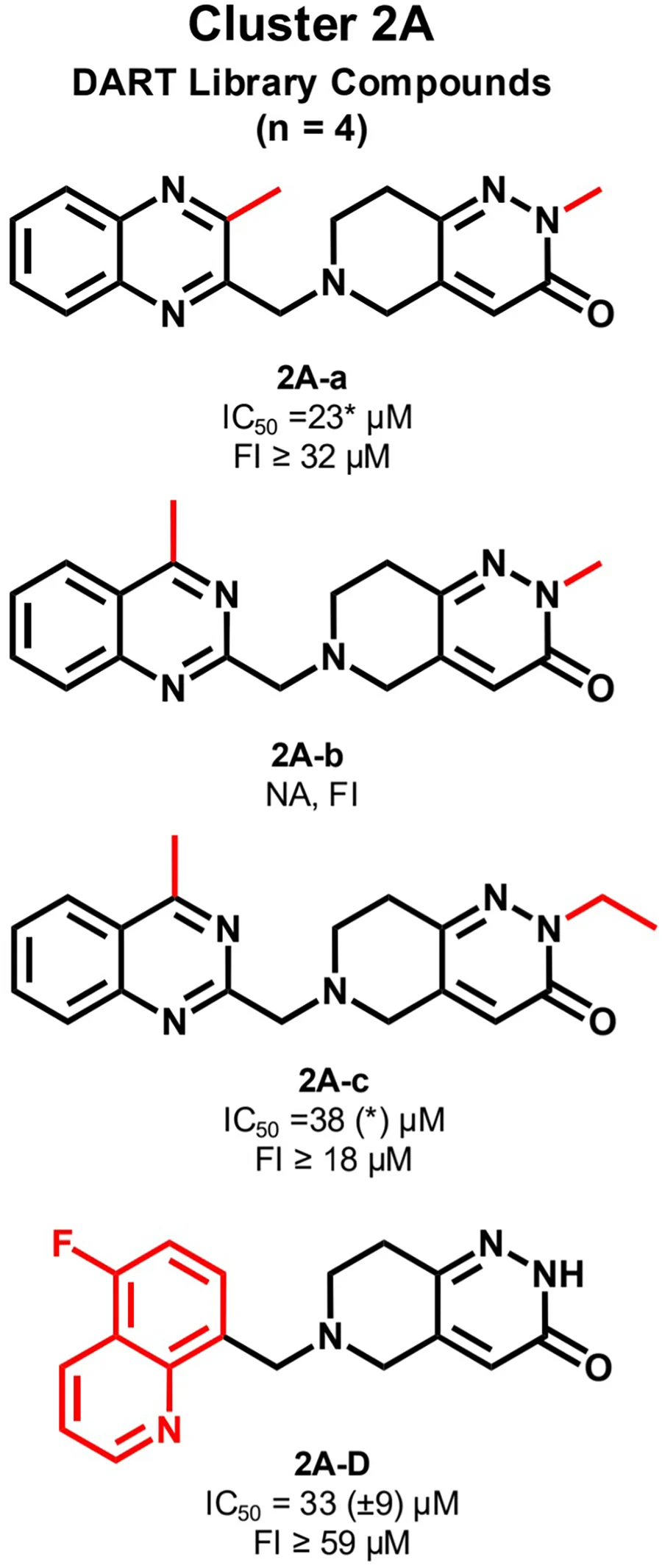
Structures of compounds from cluster 2A. Colors and * are as in Figure 5.

**Figure 7:**
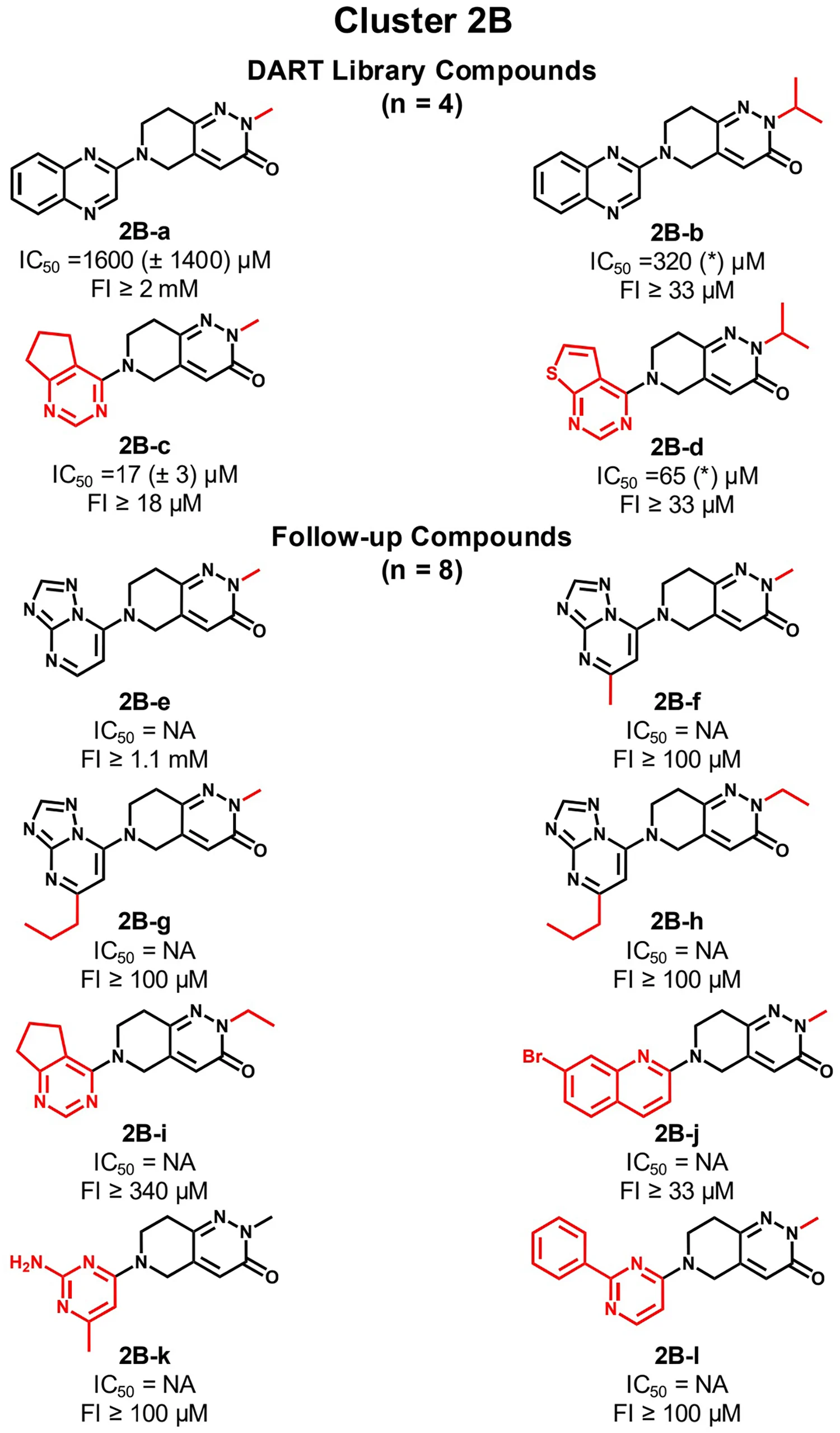
Structures of compounds from cluster 2B. The core scaffold is in black, the varied substituents are in red.

**Figure 8:**
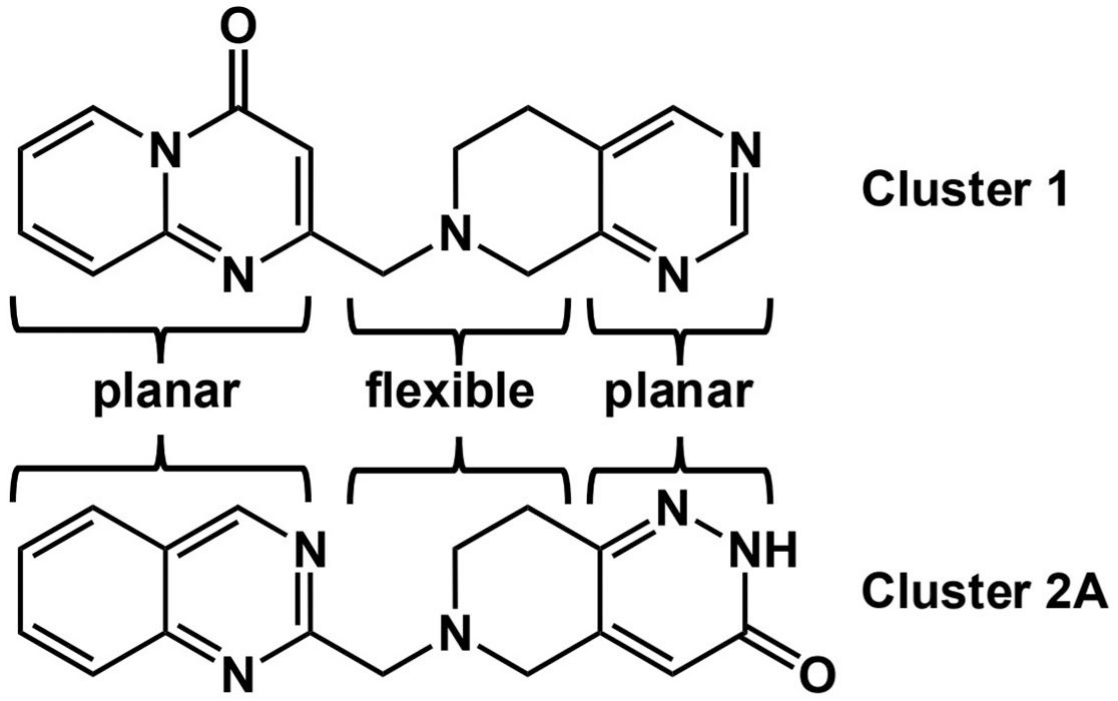
Comparison of structures of compounds from clusters 1 and 2A.

**Figure 9:**
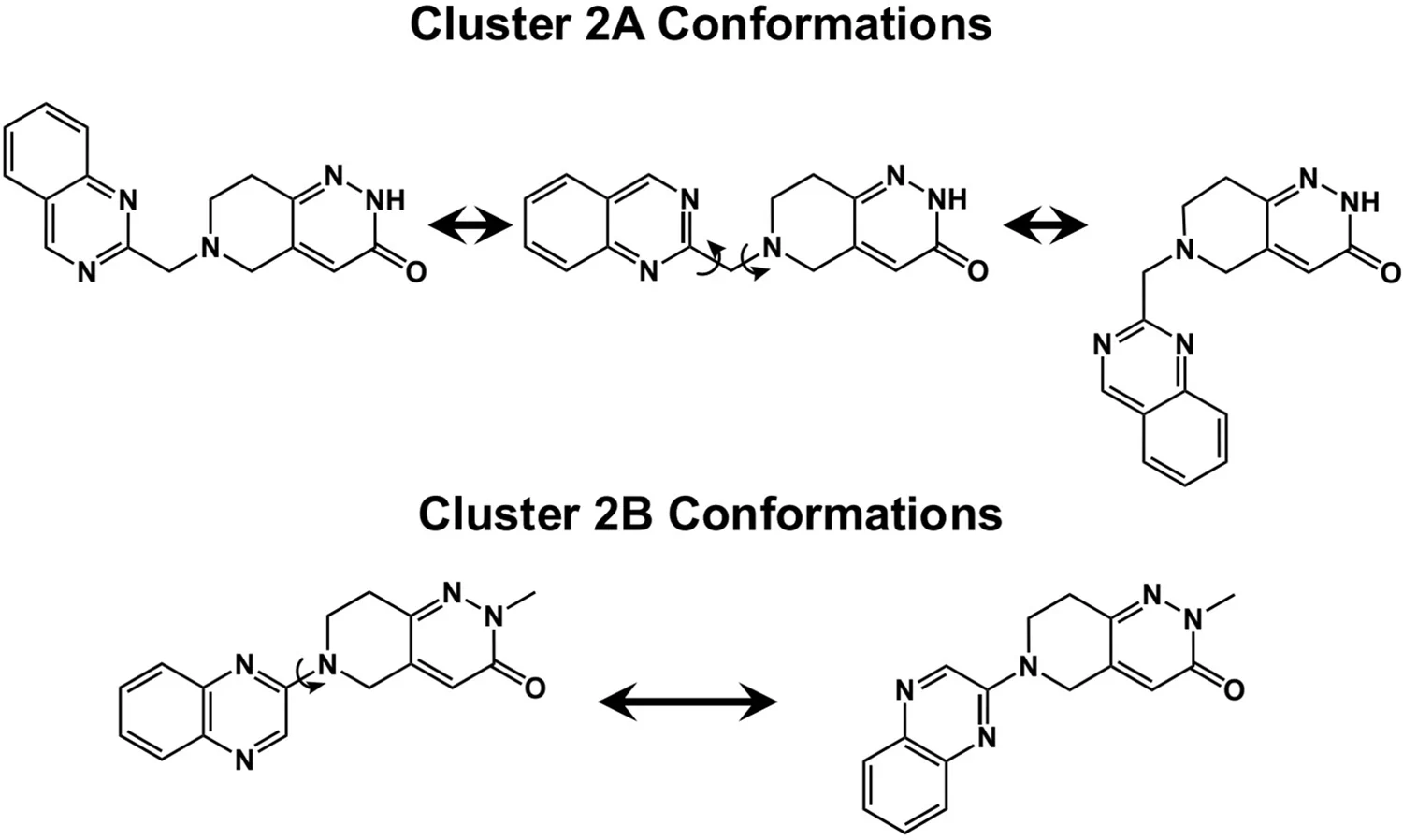
Conformations of cluster 2A and 2B core scaffolds.

There are noticeable structural similarities between compounds from Clusters 1 and 2A (Figure 9). The structures have a similar core scaffold of two biheterocyclic rings connected by a methylene. Specifically, they share a tetrahydropyridine ring in one of the core heterocycles. The scaffolds also share planarity in the left-hand heterocycle and the righthalf of the right-hand heterocycle. The methylene link between the two core heterocycles of the molecules is attached to the nitrogen of the tetrahydropyridine, giving Cluster 1 and Cluster 2A molecules very similar flexibilities and connectivity. (We elected to include Cluster 2A with Cluster 2B due to the identical heterocycle on the right half on these molecules. However, Cluster 2A could have been included in Cluster 1.).

Cluster 3 compounds are characterized by containing 2-methoxy-5-amino pyridine aromatic amide at the nitrogen, while Cluster 4 has a benzyl-6-(piperazin-1-yl) pyridin-3-amine (Figure 10). The most promising compound from Cluster 4, 4-a (IC_50_ = 38 µM), has a substituted benzene ring (a bromine in the para position and a fluorine at the meta position). Unfortunately, most of the compounds from Cluster 3 and all from Cluster 4 presented too much fluorescence interference to be considered for follow up experiments (Table 1).

**Figure 10:**
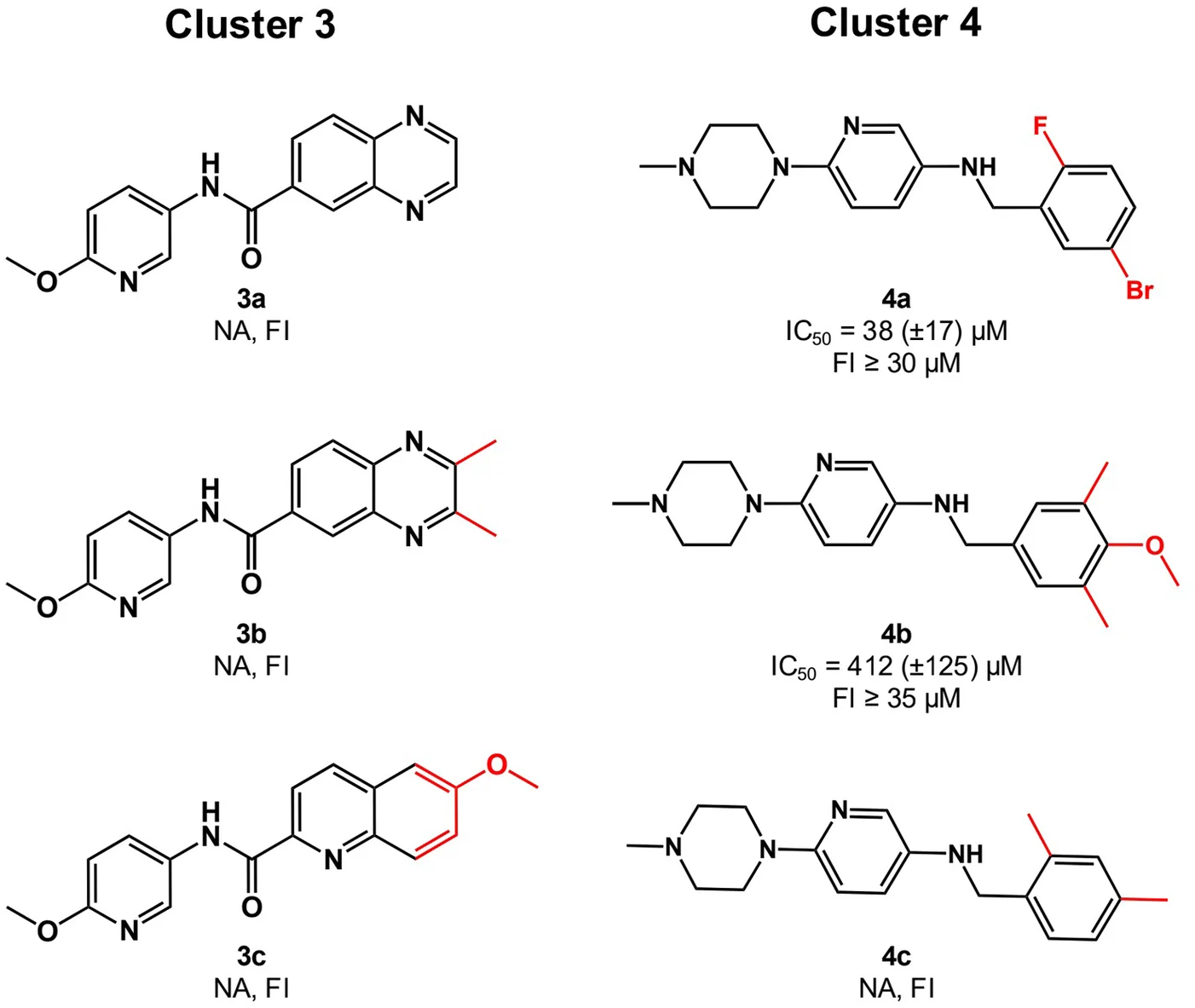
Structures of compounds from clusters 3 and 4. The * symbol represents an undefined confidence interval (CI).

## Discussion

There is a critical unmet need for novel antibiotics against MTB, exacerbated now due to the COVID-19 pandemic. The poor success in exploiting novel targets suggests a reinvestigation of targets of current and previously effective drugs. Targeting transcription has been a largely effective antibacterial strategy, as suggested by the success of both rifampin and fidaxomicin (19). Targeting transcription factors, such as CarD, may represent a new approach to inhibiting a proven anti-TB target that can compromise the pathogen by altering the stabilization of the RPo. In MTB, the gene product of CarD is required not only for persistence, but it has also been identified as an essential protein *in vitro* and *in vivo* during normal growth conditions as well as under genotoxic stress and nutrient deprivation (21–23). Therefore, CarD represents a novel potential target for therapeutic treatment against tuberculosis and potentially other clinically relevant pathogens.

Previously, a CarD FP assay that probes the interaction between CarD and the RPo was developed and used to identify compounds from a 23,320 compound library (MB24 K) (29). The Z-factor was 0.73, demonstrating that the CarD FP assay is sufficiently robust to use in an HTS format. Screening a repurposing library for inhibitors of the CarD•RNAP interaction offers a promising alternative pathway for drug development. Typically, the timeline for bringing new drugs from discovery to market, following the United States Food and Drug Administration (FDA) approval, spans approximately 20 years. Repurposing candidates, which are already FDA approved or have undergone several stages of clinical development, are generally known to possess well established safety and pharmacological profiles (26). Screening drug repurposing libraries provides benefits such as a reduction of time, effort, and cost associated with discovering new therapeutics. This method offers the potential for the fastest transition from research to clinical impact, facilitating their integration into healthcare.

The drug repurposing screen primarily served to contextualize assay performance and highlight potential sources of false positive signals. Although a small number of compounds initially appeared active, follow-up studies demonstrated that their effects were largely attributable to fluorescence interference or indirect mechanisms such as DNA binding. For example, GO-203, a peptide that has been implicated in inhibiting protein-protein interactions of a glycoprotein known as mucin 1 (MUC1), was shown to interact with DNA through its polycationic motif (31, 32). Here it was determined that GO-203 can thereby indirectly disrupt CarD•RNAP association. These findings emphasize the importance of orthogonal validation strategies when interpreting results from fluorescence-based assays. In contrast, screening of the DART library enabled the identification of a chemically diverse set of compounds with reproducible activity and no evidence of DNA binding or RNAP inhibition in orthogonal assays. While fluorescence interference remains a limitation, these compounds represent a more promising starting point for the development of direct inhibitors of the CarD•RNAP interaction.

Approximately 83,000 compounds from the DART Neurosciences library were successfully screened with our CarD FP assay, and ∼300 potential hits advanced to concentration response testing. We used the clustering tool in DataWarrior, which applies a hierarchical, bottom-up method, to group similar compounds. Compounds were grouped based on Tanimoto similarity, a common metric that compares chemical structures and gives a score from 0 to 1—where 1 means the structures are identical. Compounds with a similarity of 79% or higher were clustered together, resulting in four main clusters containing 27 compounds that were prioritized for follow-up. This analysis identified four primary clusters comprising a total of 27 compounds that were prioritized for further testing. Fresh samples of 22 of the clustered hits were purchased for confirmation and control testing. We encountered significant fluorescence interference with most of the compounds during the IC_50_ experiments for fresh samples. Accordingly, we selected only those compounds that demonstrated inhibitory activity prior to the onset of interference. Unfortunately, analogs were also showing significant fluorescence interference, making them not suitable for further analysis.

Many of the compounds from Clusters 1 and 2 had the most reliable IC_50_ data and shared similar structures. We then performed control screening to identify any false positives, compounds that indirectly interfere with CarD binding to RNAP•DNA, e.g., compounds that bind to DNA or bind to RNAP but block DNA binding. Notably, results from the DNA binding (FID) and *in vitro* transcription (MGA) assays indicated that none of the compounds targeted either RNAP or the DNA oligonucleotides, consistent with disruption of the CarD•RNAP interaction, although direct binding remains to be established. Twelve analogs (4 from Cluster 1 and 8 from Cluster 2B) were purchased, but none of these had any significant activity.

The evaluation of the 22 compounds revealed a range of antimicrobial activities (MABA MIC_90_, Table 1). MIC₉₀ values fell between 1.4 µg/mL (4.3 µM) and >100 µg/mL (310.3 µM), indicating moderate to low potency across the panel. Notably, the 2B series compounds stood out for their relatively favorable balance between antibacterial activity and cytotoxicity. Compound 2B-d, for instance, demonstrated an MIC₉₀ of 1.4 µg/mL (4.3 µM), placing it among the most potent in the series. Although its MBC₉₉ value is 47 µg/mL (143 µM), lower MBC potency is often observed even among bactericidal compounds, this suggests that while 2B-d may not exhibit strong bactericidal activity at the tested concentrations, its overall performance remains notable relative to the broader dataset. Importantly, biochemical potency did not directly correlate with whole-cell activity, as 2B-d showed weak FP activity (IC₅₀ = 65 µM) despite its strong antibacterial activity. This discrepancy may reflect differences in permeability, efflux, or target accessibility. Additionally, we cannot yet rule out that whole-cell activity may also arise from mechanisms unrelated to CarD•RNAP inhibition.

2B-d also exhibited a Vero cell TC₅₀ of 24 μM. While this indicates a level of mammalian toxicity, the calculated selectivity index (TC₅₀/MIC₉₀ = 5.6) suggests that a moderate safety margin is achievable. It is important to state that 2B-d can be a phenotypic antibacterial hit with relatively favorable MIC activity within the series, but whose cytotoxicity and low biochemical potency are obstacles that need to be addressed for it to progress as a CarD•RNAP inhibitor. Other compounds in the 2B subgroup (e.g., 2B-a, 2B-b, and 2B-c) also showed MIC₉₀ values 40–70 µg/mL and >100 µM Vero cell toxicities for 2B-a and c. The consistently high MBC₉₉ values across the dataset (>100 µg/mL for nearly all compounds) point to a common limitation in bactericidal activity, underscoring the need for structural modifications to enhance killing efficacy without increasing host cell toxicity.

As seen in Figure 8, Clusters 1 & 2A, which have the best CarD inhibitory activities, have striking structural similarities and could be combined into a single chemotype of 15 compounds. Given that 8 of these compounds have IC_50_s < 50 µM (one is < 10 µM) and 6 have MABA MIC_90_ values <100 µg/mL, this chemotype provides a starting point for future investigation of structure-activity relationships, although the modest biochemical potency, fluorescence interference, and low activity among the tested analogs currently preclude conventional lead optimization. Importantly, direct binding to either CarD or RNAP should be determined. Despite the orthogonal validation performed in this study, an important limitation is that direct target engagement has not yet been established. The FID and *in vitro* transcription assays provide no evidence that the identified compounds bind DNA or directly inhibit RNAP, supporting a mechanism distinct from these alternative modes of action. However, these results do not by themselves demonstrate direct binding to CarD or establish whether the compounds interact with CarD itself or with the CarD•RNAP interface. Thus, the precise molecular basis of inhibition remains to be determined. Future studies using complementary biophysical and structural approaches, including NMR spectroscopy and x-ray crystallography, will be important to confirm direct target engagement, define the binding site, and elucidate the structural basis of inhibition. Obtaining analogs containing more diverse substitutions at the R positions, including polar substituents (OH, NH_2_, OMe, etc.), and extension of the methylene linker should be probed.

In conclusion, we have successfully screened two distinct compound libraries against a novel mechanism of inhibition of the MTB RNA polymerase. The drug repurposing screen validated the assay for targeting the CarD•RNAP interaction and highlighted important assay limitations and indirect mechanisms of inhibition. The DART screening campaign provided a high-throughput discovery and de-risking approach that identified chemical matter capable of inhibiting the CarD•RNAP FP assay while excluding common alternative mechanisms, including DNA binding and direct RNAP inhibition. The identification of chemical matter through a PPI-directed screening strategy is consistent with the growing interest in targeting protein–protein interactions in MTB, although the present compounds require direct target-engagement studies before their mechanism can be assigned to CarD or the CarD•RNAP interface (36).

## Methods

### Protein manipulation, purification, and labeling

CarD-BODIPY-FL and RNAP were prepared as previously described with some modifications (29). For CarD-BODIPY-FL, 1 L of BL21(DE3) *E. coli* transformed with pET19bbps-CarD plasmid was grown to an OD_600_ of 0.6 in 2xTY. The cells were induced with 1 mM IPTG for 4 h at 28 °C, and then harvested to produce a pellet. Cells were resuspended in 20 mL of lysis buffer (20 mM Tris HCl (pH 8.0), 500 mM NaCl, 0.1 mM EDTA, with 2 mM PMSF, 1× Roche Protease cocktail, 1 mg/mL lysozyme, and 200 U DNase I, 5% glycerol (v/v), 5 mM *β*-mercaptoethanol (*β*ME)). After sonication (2 s off, 3 s on, 1 min×3 times), lysate was cleared of cell debris by centrifugation at 30,000×*g* for 45 min at 4 °C. Lysate was sterile filtered and injected into a 1 mL HisTrap HP column. CarD was eluted by stepwise gradient to 500 mM imidazole and dialyzed overnight into labeling buffer [100 mM Tris HCl (pH 7.5 at 25 °C), 100 mM NaCl, and 1 mM EDTA] at 4 °C. A tenfold molar excess of BODIPY-FL iodoacetamide{[*N*-(4,4-difluoro-5,7-dimethyl-4-bora-3a,4a-diaza-s-indacene-3-yl) methyl] iodoacetamide, Thermo Fisher Scientific} suspended in DMF was added dropwise to the CarD solution with stirring, allowing the reaction to proceed for 4 h at 24 °C. The reaction was quenched with excess *β*ME, and then *PreScission* protease was added to the mixture which was dialyzed overnight in SEC Buffer [20 mM Tris HCl (pH 8.0), 300 mM NaCl, 0.1 mM EDTA, 5% glycerol, and 2 mM DTT]. Excess BODIPY-FL and *PreScission* protease were removed from the incubation mixture by applying the mixture to a Superdex 200 10/300 GL column equilibrated with SEC buffer. The resulting labeled CarD was stored at −80 °C. To determine the degree of labeling, the absorbance at 280 nm and at the wavelength max (504 nm) of the dye were measured using a UV-Vis transparent plate (BD Falcon 384-well plate). The absorbance of the protein was calculated using equation A_prot_=A_280_ – A_504_ × CF_280_. A_prot_ is the absorbance at 280, *ε*_prot_ is the molar extinction coefficient of CarD, and CF is the correction factor for the BODIPY-FL absorbance at 280 nm (provided by the company, BODIPY-FL CF_280_ = 0.04). Using the A_prot_ value, protein and BODIPY-FL (BDFL) molar extinction coefficients (*ε*_prot_ = 19,940 M^−1^cm^−1^ and *ε*_BDFL_ = 68,000 M^−1^cm^−1^, respectively), the concentrations of the total protein and labeled protein were determined using the equations: c_prot_=A_prot_/*ε*_prot_ × pathlength and c_BDFL_=A_504_/*ε*_BDFL_ × pathlength, respectively. To determine the percentage of labeling, the equation (c_BDFL_/c_prot_) × 100 was used. Extents of labeling between 60%–70% were obtained for our BODIPY-FL labeled CarD purifications.

For the SigA purification, a 1 L culture was grown at 37 °C to OD_600_ = 0.4–0.6, which was then induced with 1 mM IPTG for 16–20 h at 16 °C. Cells were harvested and resuspended in 15 mL SigA cell lysis/Bind Buffer [10 mM Tris-HCl (pH 8 at 4 °C), 0.5 M NaCl, 5% glycerol, and 5 mM *β*-ME, 2 mM PMSF]. After sonicating the cells (13 s intervals on, 7 times, with 90 s of rest in between), the lysate was cleared of cell debris by centrifugation at 21,000×g for 40 min at 4 °C. The sterile filtered supernatant was applied into a 1 mL HisTrap HP column. SigA was eluted with a stepwise gradient (0–500 mM imidazole). Eluted protein was applied to a Sephacryl S-200HR column pre-equilibrated with SigA running buffer (10 mM Tris-HCl, pH 8 at 4 °C, 200 mM NaCl, 5% glycerol, 0.1 mM EDTA, 5 mM DTT) for size-exclusion chromatography. Fractions containing SigA, determined from the UV chromatographic trace and confirmed by SDS-PAGE, were flash-frozen in liquid nitrogen and stored at −80 °C.

### Card fluorescence polarization assay

The fluorescence polarization (FP) assay was performed in a final volume of 20 µL. The reaction mixture contained FP buffer [20 mM Tris HCl, pH 7.9 at 25 °C, 150 mM potassium glutamate (KGlu), 10 mM MgCl_2_, 0.1 mM EDTA, 0.01% Triton X-100, 5 mM DTT], “native” rrnAP3 DNA fragment at a final concentration of 600 nM, CarD-BODIPY-FL at 5 nM, SigA at an excess concentration of 40 nM, and WT MTB RNAP at 20 nM. IC_50_ values were obtained by testing compounds starting at 20 µM followed by serial 1.8× dilutions. Assay components were incubated at 25 °C for 4 h before being read in a BioTek Synergy H1 Hybrid Multi-mode plate reader (excitation: 485/20 nm, emission: 528/20 nm). Fluorescence millipolarization (mP) was calculated using the equation mP = [(F_II_ - F_⊥_)/(F_II +_ F_⊥_)]*1000, where F_II_ represents the parallel fluorescence intensity and F_⊥_ indicates the perpendicular fluorescence intensity. FP values vs. compound concentrations were fit to a four-parameter sigmoidal dose–response with top and bottom limits unconstrained for each curve.

### High throughput screen (HTS): drug repurposing library (DRL)

A high throughput screen of the Drug Repurposing Library (30) (4,719 compounds) was conducted using the CarD-BODIPY-FL FP assay described above with the modification that the [rrnAP3] was 300 nM. A volume of 10 µL of 2 × MTB RNAP/ CarD-BODIPY-FL/SigA mixture was added to the 384-well plate using a multidrop dispenser (Thermo Fisher Scientific) followed by 20 nL of 2 mM compound stock solution in DMSO using an Echo 655 Liquid Handler to obtain a final compound concentration of 20 µM. Compounds were incubated with the MTB RNAP/CarD-BODIPY-FL/SigA mixture for 20 min and then read to determine fluorescence interference prior to the addition of 10 µL of 2 × DNA mix. DMSO (20 nL) without compound was added in wells representative of the negative control and for positive controls DNA was replaced with buffer solution. Reactions were allowed to come to equilibrium at 25 °C for 4 h and then read using a BioTek Synergy H1 Hybrid Multi-mode plate reader (excitation: 485/20 nm, emission: 528/20 nm). The Z-factor (Z) was calculated using the equation Z = 1- [3(*σ*_P_ + *σ*_N_)/ (µ_P_ + µ_N_)], where µ_P_ and µ_N_ are the means and *σ*_P_ and *σ*_N_ are the standard deviations of the positive and negative controls respectively.

### High throughput screen (HTS): DART neurosciences library

A high throughput screen of the DART Neurosciences library (82,516 compounds) was conducted using the CarD-BODIPY-FL FP assay described above with the modification that the [rrnAP3] was 300 nM. A volume of 10 µL of 2 × MTB RNAP/ CarD-BODIPY-FL/SigA mixture was added to the 384-well plate using a multidrop dispenser (Thermo Fisher Scientific) followed by 20 nL of compound stock solution in DMSO using an Echo 655 Liquid Handler to obtain a final compound concentration of 20 µM or to obtain serial dilutions for IC_50_. Compounds were incubated with the MTB RNAP/CarD-BODIPY-FL/SigA mixture for 20 min and then read to determine fluorescence interference prior to the addition of 10 µL of 2 × DNA mix. DMSO (20 nL) without compound was added in wells representative of the negative control and for positive controls DNA was replaced with buffer solution. Reactions were allowed to come to equilibrium at 25 °C for 4 h and then read using a Pherastar plate reader with a green filter polarization cube (485/20 ex and 528/20 em). The Z-factor (Z) was calculated in the same manner as for the repurposing library screen.

### Solubility/turbidity analysis

Compounds were tested in reaction buffer. Two µL of compound was mixed with 18 µL of 1× buffer in a 384-well polystyrene UV transparent plate (Corning 3680). The plate was incubated for 4 h at 25° C and measured at an absorbance of 620 nm.

### Fluorescent intercalator displacement (FID) analysis

The binding of compounds to DNA was determined by fluorescence intercalator displacement (FID). The 60 bp FID-lacUV5 probe was hybridized by mixing the top and bottom oligonucleotides 1:1 after suspending in TE/NaCl buffer (10 mM Tris-HCl, 1 mM EDTA, 50 mM NaCl, pH = 8.0). The oligonucleotides were annealed in a water bath by heating at 94° C for 2 min, followed by 7°C for 2 min, and then were allowed to slowly cool to room temperature. In a 384 plate, 14 µL of 64.3 µM ethidium bromide in Tris buffer (0.1 M Tris HCl, 0.1 M NaCl, pH = 8.0) was added to all wells. Then, 2 µL of 15 µM FID lacUV5 probe in TE/NaCl buffer or TE/NaCl buffer (background fluorescence control) was added, followed by the addition of 4 µL test compound in 10% DMSO Tris buffer (10% DMSO Tris buffer for 100% fluorescence control). Peptides were each tested at four different concentrations (1 mM–100 µM or 100–10 µM). The plates were protected from light and allowed to equilibrate for 30 min. Fluorescence was measured after incubation (excitation/emission=545/595 nm). The background fluorescence was subtracted from all data. The percent fluorescence inhibition was calculated according to % EtBr fluorescence=[(F_100%_- *F*_test_)/(F_100%_)] *100, where F_test_ is the average fluorescence obtained in the presence of test compound and F_100%_ is the average fluorescence of the 100% fluorescence control.

### Malachite green aptamer assay (MGA)

The activity of MTB RNAP was measured via plasmid-based, *in vitro* transcription using the template plasmid pMGA4-Mt-rrnA3-SynBx3, as previously reported (29). A 50 μL reaction was prepared in Corning 96 Well Half Area Black Flat Bottom Polystyrene NBS™ microplates. In each well, 23 μL of a 2× RNAP reaction mix solution [80 mM Tris-HCl (pH = 7.5 at 23° C), 100 mM KCl, 20 mM MgCl_2_, 0.02% Triton X-100, 16 mM DTT, 40 nM RNAP, 120 nM SigA] was added, followed by 2 μL of compound in DMSO, and then by 25 μL of a 2× DNA-NTP solution (40nM pMGA4-Mt-rrnA3-SynBx3, 4 mM NTPs). As controls, either DMSO (positive control) or 1 µM of rifampin were used instead of compound. The plate was incubated at 37° C for 3 h, then the reaction was stopped by incubating on ice and adding 50 μL of cold 60 μM malachite green in water. After 10 min on ice, malachite green fluorescence was detected at excitation and emission wavelengths of 628 nm and 660 nm, respectively. The % fluorescence inhibition (FI) was calculated by dividing the fluorescence value of each concentration by the positive control fluorescence value and multiplying it by 100.

### Minimum inhibitory concentration (MIC_90_) and minimum bactericidal concentration (MBC_99_)

All compounds were evaluated for MIC vs. MTB H_37_R_V_ ATCC 27294 using the MABA (33). Activity against non-replicating cultures of *M. tuberculosis* H37Rv transformed with lux ABCDE (34) was determined using the LORA (33). The MIC was defined as the lowest concentration effecting a reduction in luminescence of ≥ 90% relative to untreated bacteria controls.

The MBC was determined by incubating test compounds with *M. tuberculosis* H37Rv for one week at 37° C (35). Cultures were centrifuged, and pellets were resuspended and serially diluted in phosphate buffered saline and then plated on Middlebrook 7H11 agar. Colonies were enumerated following a 2–3 week incubation at 37° C. Time-kill studies were performed with *M. tuberculosis* transformed with luxABCDE (34) and determining luminescence at selected time intervals. The MBC at a given time point was defined as the lowest tested concentration effecting a reduction in luminescence of ≥ 99% relative to that observed prior to the addition of test compounds.

### Vero cell toxicity

Cytotoxicity for a mammalian cell line was determined by exposing Vero cells (ATCC CRL-81) to serial dilutions of test compounds for 72 h, followed by addition of resazurin and measurement of fluorescence after a four hr incubation (35). The TC_50_ was defined as the concentration effecting a reduction in fluorescence of 50% relative to untreated cells.

## Data Availability

The datasets presented in this study can be found in online repositories. The names of the repository/repositories and accession number(s) can be found in the article/Supplementary Material.
