# Supplementary material for: High-throughput screening to identify inhibitors of the MTB CARD•RNA polymerase protein-protein interaction
Source: Front Tuberc. 2026 Oct 2;4:1937953. doi: 10.3389/ftubr.2026.1937953 (PMC13672193; doi:10.3389/ftubr.2026.1937953)
Supplement: Supplementary file 1 [file Supplementaryfile1.docx]

***Velazquez et al. Supplementary Material***

| **Product Name** | **Target** | **Molecular Weight (MW)** | **ALogP** | **H-Bond Acceptors (HBA)** | **H-Bond Donors (HBD)** | **Rotatable Bonds** |
| --- | --- | --- | --- | --- | --- | --- |
| Benztropine mesylate | Dopamine Receptor | 404 | 3.4 | 1 | 0 | 4 |
| Fidaxomicin | DNA/RNA Synthesis | 105 | 7.7 | 11 | 7 | 15 |
| Fenbufen | COX | 25 | 2.9 | 2 | 0 | 5 |
| Tolmetin | Immunology & Inflammation | 25 | 3.3 | 2 | 0 | 4 |
| Octenidine·2HCl | Anti-infection | 624 | 10.6 | 2 | 0 | 25 |
| NH125 | CaMK | 52 | 6 | 0 | 0 | 17 |
| GO-203 | Others | 2426 | −21.0 | 19 | 41 | 98 |

Supplementary Figure 1. DRL Library Screen Flowchart (A) and Hits Table (B).

Supplemental Figure 2. GO-203 and Component Peptides Structures and Plots of Activities. (A) & (B) GO-203, (C) & (D) R9 peptide, (E) & (F) CQC peptide. CarD FP (red) is on the left Y-axis and parallel fluorescence intensity (green) is on the right Y-axis. IC_50_ values are noted. Data points are the mean of three determinations, and the errors bars are the standard deviations of those means.

400

**IC50 =7 (± 2) µM**

Millipolarization Units (mP)

## Z1139229489

150000

400

**IC50 = 25 (*) µM**

Millipolarization Units (mP)

## Z1139231794

100000

300

200

100

100000

50000

**Parallel Intensity**

350

300

80000

60000

**Parallel Intensity**

40000

0	0

-6	-5	-4	-3

#### Log (M)

250

-6	-5	-4	-3

#### Log (M)

20000

 Z1139229489 mP

 Z1139229489 mP

 Z1139231794 mP

 Z1139231794 mP

 Z1139229489 FI

 Z1139231794 FI

400

**IC50 =30 (± 25) µM**

Millipolarization Units (mP)

## Z1139232215

80000

400

## Z1139229798

100000

**Parallel Intensity**

350

300

60000

40000

**Parallel Intensity**

20000

300

200

100

50000

250

Millipolarization Units (mP)

0

-6	-5	-4	-3

#### Log (M)

0	0

-6	-5	-4	-3

#### Log (M)

 Z1139232215 mP

**IC50 = 36 (± 13) µM**

 Z1139232215 mP

 Z1139229798 mP

 Z1139229798 mP

 Z1139232215 FI

 Z1139229798 FI

400

Millipolarization Units (mP)

350

300

250

### Z1142760457

-6	-5	-4	-3

**IC50 = 117 (*) µM**

100000

80000

Parallel Intensity

60000

40000

400

300

Millipolarization Units (mP)

200

100

0

## Z1139230008

-6	-5	-4	-3

#### Log (M)

100000

80000

**Parallel Intensity**

60000

40000

20000

0

Log (M)

 Z1142760457 mP

Z1142760457 mP

 Z1139230008 mP

 Z1139230008 mP

400

Millipolarization Units (mP)

 Z1142760457 FI

## Z1139233224

Millipolarization Units (mP)

150000

400

 Z1139230008 FI

## Z1139229390

200000

300

200

100

0

100000

50000

**Parallel Intensity**

0

300

200

100

0

-6	-5	-4

150000

100000

**Parallel Intensity**

50000

0

-3

-7	-6	-5	-4	-3

#### Log (M)

**Log (M)**

 Z1139233224 mP

 Z1139233224 mP

 Z1139229390 mP

 Z1139229390 mP

 Z1139233224 FI

 Z1139229390 FI

400

Millipolarization Units (mP)

300

200

100

## Z1272713291

150000

**IC50 = 23 (*) µM**

**Parallel Intensity**

100000

50000

400

350

**IC50 = 33 (± 9) µM**

Millipolarization Units (mP)

300

## Z1732356336

100000

80000

**Parallel Intensity**

60000

40000

20000

0	^0^	250	0

-7	-6	-5	-4	-3

#### Log (M)

-6	-5	-4	-3

#### Log (M)

 Z1272713291 mP

 Z1272713291 mP

 Z1732356336 mP

 Z1732356336 mP

 Z1272713291 FI Z1732356336 FI

400

Millipolarization Units (mP)

350

300

250

## Z1272699796

100000

**IC50 = 38 (*) µM**

**Parallel Intensity**

80000

60000

40000

**400**

**350**

**Millipolarization Units (mP)**

**300**

**250**

## Z1272711377

**100000**

**80000**

**Parallel Intensity**

**60000**

**40000**

-6	-5	-4

#### Log (M)

**-6.5	-6.0	-5.5	-5.0	-4.5	-4.0**

#### Log (M)

 Z1272699796 mP

 Z1272699796 mP

 Z1272711377 mP

 Z1272711377 mP

400

Millipolarization Units (mP)

300

200

100

0

 Z1272699796 FI

## Z1272698803

**IC50 = 17 (± 3) µM**

-6	-5	-4	-3

#### Log (M)

80000

60000

**Parallel Intensity**

40000

20000

0

380

360

Millipolarization Units (mP)

340

320

300

280

 Z1272711377 FI

## Z1272698415

**IC50 =1.6 mM (± 1.4) µM**

-6	-5	-4	-3

#### Log (M)

80000

60000

**Parallel Intensity**

40000

20000

 Z1272698803 mP

 Z1272698803 mP

 Z1272698415 mP

 Z1272698415 mP

400

Millipolarization Units (mP)

350

300

250

 Z1272698803 FI

## Z1272699303

-6	-5	-4	-3

#### Log (M)

100000

80000

**Parallel Intensity**

60000

40000

400

350

Millipolarization Units (mP)

300

250

 Z1272698415 FI

## Z1272699295

-6	-5	-4	-3

#### Log (M)

15000

10000

**Parallel Intensity**

5000

 Z1272699303 mP

 Z1272699303 mP

 Z1272699295 mP

 Z1272699295 mP

 Z1272699303 FI

 Z1272699295 F

Supplemental Figure 3. Plots of CarD Polarization and Parallel Fluorescence For DART Hits

Supplementary Figure 4. Control Screen Plots for DART Hits. (A) Turbidity (OD620) vs. Concentration, (B) Fluorescent Intercalator Displacement (% Ethidium Bromide Fluorescence) vs. Concentration, and (C) In vitro transcription inhibition (% Malachite Green Fluorescence) vs. Concentration. Data bars are the means of three determinations, and the errors bars are the standard deviations of those mean.
